# Chronic ET_A_ antagonist reverses hypertension and impairment of structure and function of peripheral small arteries in aortic stiffening

**DOI:** 10.1038/s41598-018-20439-5

**Published:** 2018-02-15

**Authors:** Xiaomei Guo, Huan Chen, Ling Han, Stephan Haulon, Ghassan S. Kassab

**Affiliations:** 1 California Medical Innovations Institute, San Diego, California USA; 2 Aortic Center, Hôpital Marie Lannelongue, Le pLessis Robinson, Université Paris Sud, Clemenceau, 91400 Orsay France

## Abstract

Arterial stiffness may contribute to the pathogenesis of hypertension. The goal of this study is to elucidate the role of Endothelin-1 (ET-1) in aortic stiffening-induced hypertension through ET_A_ receptor activation. An increase in aortic stiffness was created by use of a non-constrictive restraint, NCR on the abdominal aortic surface. A group of rats underwent aortic NCR or sham operation for 12 weeks and were then treated with ET_A_ receptor antagonist BQ-123 for 3 weeks. We found that 12 weeks of aortic NCR significantly increased pulse and mean pressure and altered peripheral flow pattern, accompanied by an increased serum ET-1 level (p < 0.05). The increase in aortic stiffness (evidenced by an elevated pulse wave velocity) caused hypertrophic structural remodeling and decreased arterial compliance, along with an impaired endothelial function in peripheral small arteries. BQ-123 treatment only partially attenuated peripheral arterial hypertrophy and restored arterial compliance, but completely recovered endothelium function, and consequently restored local flow and lowered blood pressure. Our findings underscore the hemodynamic coupling between aortic stiffening and peripheral arterial vessels and flow dynamics through an ET_A_-dependent mechanism. ET_A_ receptor blockade may have therapeutic potential for improving peripheral vessel structure and function in the treatment of aortic stiffness-induced hypertension.

## Introduction

Increased central arterial stiffness often precedes all-cause mortality and total cardiovascular events including aging, myocardial infarction, diabetes, atherosclerosis, heart failure, and stroke^1–5^. Aortic stiffening is associated with changes in blood pressure profile characterized by increase in systolic pressure and/or increased pulse pressure^6,7^. The current knowledge supports a two-way interaction where increased aortic stiffness may not only be the result of hypertension, but also a cause of hypertension^8,9^. The underlying pathophysiological mechanisms for the two-way interaction, however, remain obscure. There is general agreement that the onset of hypertension is related to increased peripheral vascular resistance to blood flow^10,11^. Prior studies have confirmed that aortic stiffening increases pulsatile hemodynamic forces, which may trigger rarefaction, remodeling and increased tone in the microcirculation^12,13^. In contrast to pressure dynamics, flow dynamics of peripheral arteries in response to aortic stiffening has rarely been investigated. An alteration in local blood flow can lead to arterial structural remodeling in order to maintain homeostatic values of wall shear stress and circumferential wall stress^14^. Although stiffness-induced hemodynamic changes have been implicated in the development of hypertension^15,16^, little is known about the hemodynamic relationship linking aortic stiffening and the resulting widened pulse pressure (PP) to altered structural, mechanical and functional properties of peripheral arteries as well as impaired peripheral flow patterns. The goal of the present study is to fill this gap.

Endothelin-1 (ET-1) is a peptide produced primarily by vascular endothelial cells and is characterized as a powerful smooth muscle vasoconstrictor and mitogen^17^. It is well known that increased ET-1 levels are associated with atherosclerosis, hypertension, cardiovascular pathophysiology and renal dysfunction^18–21^. ET-1 via endothelin type A (ET_A_) receptor leads to vasoconstriction, mitogenesis, and anti-apoptotic effect with increased intracellular Ca^+2^ concentrations^22^. It has been reported that ET-1 may contribute to endothelial dysfunction and arterial hypertrophy in hypertension^23,24^. Moreover, the increased vasoconstrictor sensitivity of arteries to ET-1 in hypertension is thought to relate to the increased expression of the ET_A_ receptor protein^25^. Therefore, ET-1 receptor antagonists have been established as a first-line option for patients with pulmonary arterial hypertension^26^. Although ET-1 receptor blockade was approved to lower blood pressure in animals and patients^26,27^, there is lack of direct evidence whether blood pressure controlled by ET-1 receptor antagonists is associated with their direct effects on peripheral vascular structure and function. One of the goals of this study is to elucidate the role of ET-1 in the aortic stiffening-induced hypertension rat model through ET_A_ receptor activation.

The two major hypotheses considered in this study are: (1) Aortic stiffening results in structural and functional remodeling of peripheral small arteries and impaired regulation of local flow; and (2) Treatment with ET_A_ receptor antagonist has beneficial effects on peripheral arterial remodeling and local flow pattern to normalize blood pressure. To test these hypotheses, an increase in aortic stiffness in a normal rat model was created by use of a non-constrictive restraint, NCR (glue coating) on the external surface of abdominal aorta. The chronic administration of the specific ET_A_ receptor antagonist (BQ-123) was performed in aortic NCR animals.

## Results

Table 1 lists body weights and hemodynamics parameters before and after aortic NCR and BQ-123 treatment. The baseline of body weight, heart rate, blood pressure and peripheral blood flow were comparable in all groups. Twelve weeks of aortic NCR and 3 weeks of BQ-123 treatment had no significant influences on body weights and heart rates as compared to sham. In sham rats, no difference in blood pressure and peripheral blood flow was seen before and after aortic NCR and BQ-123 treatment. In NCR rats, the central and peripheral aortic PP, systolic blood pressure (SBP), diastolic blood pressure (DBP) and mean arterial pressure (MAP) were significantly increased when compared with pre-intervention level and sham group (P < 0.05, paired *t-*test). Chronic treatment with BQ-123 caused a significant decrease in central and peripheral aortic MAP, SBP and DBP (p < 0.05, paired *t-*test). Although the central and peripheral aortic PP after BQ-123 treatment tended to decrease, but the changes were not significantly different from the NCR group.

**Table 1:** Body weights and hemodynamics parameters before and after aortic NCR and BQ-123 treatment.

Group	Baseline	Aortic NCR	BQ-123 treatment
Body Weight (g)			
Exp	467.1 ± 14.2	627.2 ± 20.4	641.3 ± 18.9
Sham	448.4 ± 9.0	608.3 ± 9.3	626.7 ± 13.6
Heart rate (beats/min)			
Exp	364.1 ± 6.8	363.2 ± 8.9	353.0 ± 6.9
Sham	349.8 ± 16.2	360.3 ± 12.1	341.7 ± 12.2
Central aortic blood pressure (mmHg)			
Mean pressure			
Exp	111.1 ± 3.2	131.3 ± 3.4*	115.0 ± 3.8^#^
Sham	109.9 ± 4.6	114.7 ± 2.7	116.7 ± 2.7
Systolic pressure			
Exp	128.2 ± 3.7	151.8 ± 3.4*	133.3 ± 3.9^#^
Sham	129.6 ± 5.5	131.3 ± 3.2	134.1 ± 2.8
Diastolic pressure			
Exp	93.5 ± 3.2	108.5 ± 2.9*	94.8 ± 3.2^#^
Sham	92.0 ± 3.3	97.0 ± 2.8	99.3 ± 2.1
Pulse pressure			
Exp	35.3 ± 1.5	43.3 ± 1.0*	38.5 ± 1.8
Sham	37.6 ± 2.2	34.3 ± 0.7	34.8 ± 1.4
Peripheral aortic blood pressure (mmHg)			
Mean pressure			
Exp	105.2 ± 3.2	126.2 ± 3.5*	108.0 ± 4.5^#^
Sham	101.7 ± 4.7	107.3 ± 1.8	112.7 ± 2.3
Systolic pressure			
Exp	128.8 ± 3.6	152.7 ± 3.7*	133.4 ± 5.1^#^
Sham	127.0 ± 5.5	131.6 ± 2.6	135.7 ± 3.3
Diastolic pressure			
Exp	88.4 ± 3.0	102.8 ± 3.4*	87.6 ± 3.5^#^
Sham	85.7 ± 3.6	92.8 ± 2.5	95.1 ± 2.6
Pulse pressure			
Exp	40.4 ± 1.1	49.9 ± 1.1*	45.8 ± 2.1
Sham	41.3 ± 2.1	38.8 ± 1.5	40.6 ± 1.8
PWV (m/s)			
Exp	2.4 ± 0.3	4.9 ± 0.3*	4.1 ± 0.3
Sham	2.8 ± 0.1	3.1 ± 0.1	3.2 ± 0.1
Peripheral flow rate (ml/min)			
Mean flow			
Exp	0.093 ± 0.005	0.049 ± 0.004*	0.099 ± 0.008^#^
Sham	0.091 ± 0.01	0.10 ± 0.006	0.099 ± 0.01
Forward flow			
Exp	0.33 ± 0.02	0.21 ± 0.05*	0.34 ± 0.04^#^
Sham	0.30 ± 0.05	0.35 ± 0.03	0.32 ± 0.06
Reverse flow			
Exp	−0.022 ± 0.005	−0.065 ± 0.006*	−0.026 ± 0.007^#^
Sham	−0.019 ± 0.006	−0.025 ± 0.007	−0.022 ± 0.006
Reverse/forward flow ratio			
Exp	6.7 ± 1.1	30.9 ± 1.5*	7.6 ± 1.4^#^
Sham	6.3 ± 1.9	7.1 ± 1.8	6.8 ± 1.5

Values are mean ± SEM for sham and experimental (Exp) groups. *P < 0.05, when compared to baseline and sham; ^#^P < 0.05, when compared to aortic NCR (paired t-test).

Pulse wave velocity (PWV), as an index of arterial stiffness, was significantly increased by 2 fold after 12 weeks of aortic NCR when compared with pre-intervention level and sham (p < 0.01, paired *t-*test, Table 1). Following 3 weeks of BQ-123 treatment, PWV remained elevated as compared to sham (p < 0.05, paired *t-*test).

**Figure 1:**
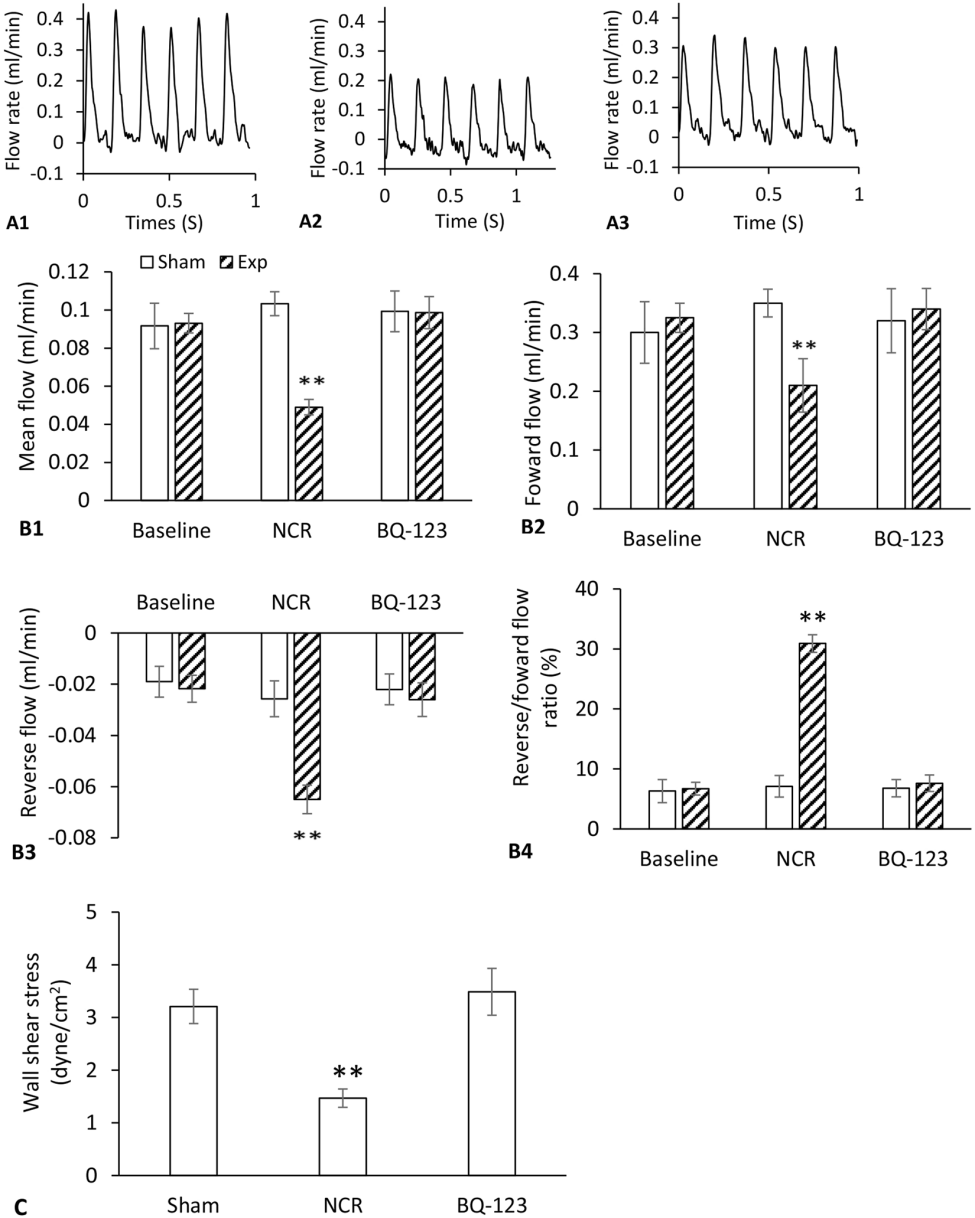
(**A**) Representation of flow waveform of peripheral small arteries with positive and negative flow peaks for sham (**A1**), aortic NCR (**A2**) and BQ-123 (**A3**) treatment groups; (**B**) Peripheral mean (**B1**), forward (**B2**), reverse (**B3**) flow rate and reverse/forward flow ratio (**B4**) for baseline, aortic NCR and BQ-123 treatment groups; (**C**) Wall shear stress (WSS) of peripheral small arteries for sham, aortic NCR and BQ-123 treatment groups. Data correspond to mean ± SEM. **P < 0.05, when aortic NCR (n = 10) compared with sham (n = 8) and BQ-123 (n = 9) groups.

The small peripheral arteries for all experimental groups exhibited a bidirectional pulse flow waveform with positive and negative peaks, consisting of the initial forward flow in systole and the secondary reverse flow in diastole (Fig. 1A). After 12 weeks of aortic NCR, peripheral mean flow and forward flow were significantly reduced by 0.5 fold, whereas reverse flow and reverse/forward (R/F) ratio were increased by 2~3 fold as compared to sham (Table 1). BQ-123 treatment completely restore the mean flow, forward and reverse flow to their pre-intervention levels (p < 0.001, paired *t-*test) as shown in Fig. 1B.

The wall shear stress (WSS) was calculated according to Equation 2. Figure 1C shows WSS in peripheral small arteries after aortic NCR and BQ-123 treatment. The WSS was significantly decreased after aortic NCR compared with sham group (p < 0.05, paired *t-*test), but its value returned to the sham level following BQ-123 treatment.

**Figure 2:**
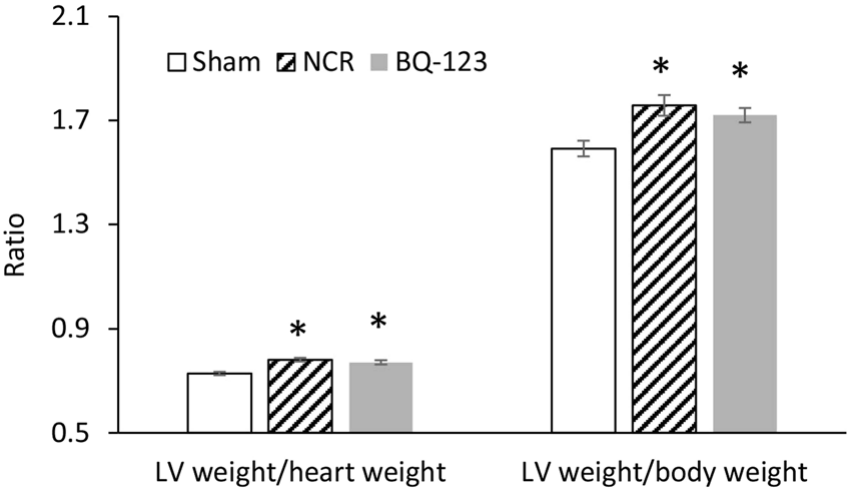
LV weight/heart weight and LV weight/body weight ratio for sham, aortic NCR and BQ-123 treatment groups. Data correspond to mean ± SEM. *P < 0.05, when aortic NCR (n = 10) or BQ-123 (n = 9) compared with sham (n = 8) group.

Heart and left ventricle (LV) weights were measured for all groups of rats. The wet weights of the hearts did not change in aortic NCR and BQ-123 treatment groups as compared to sham. Figure 2 shows LV weight/heart weight ratio (LV/HW) and LV weight/body weight (LV/BW) ratio for sham, aortic NCR and BQ-123 treatment groups. The LV/HW ratio in aortic NCR and BQ-123 treatment groups showed a significant increase when compared to sham (p < 0.05, paired *t-*test). Similar result was found for the LV/BW ratio (g/kg).

**Figure 3:**
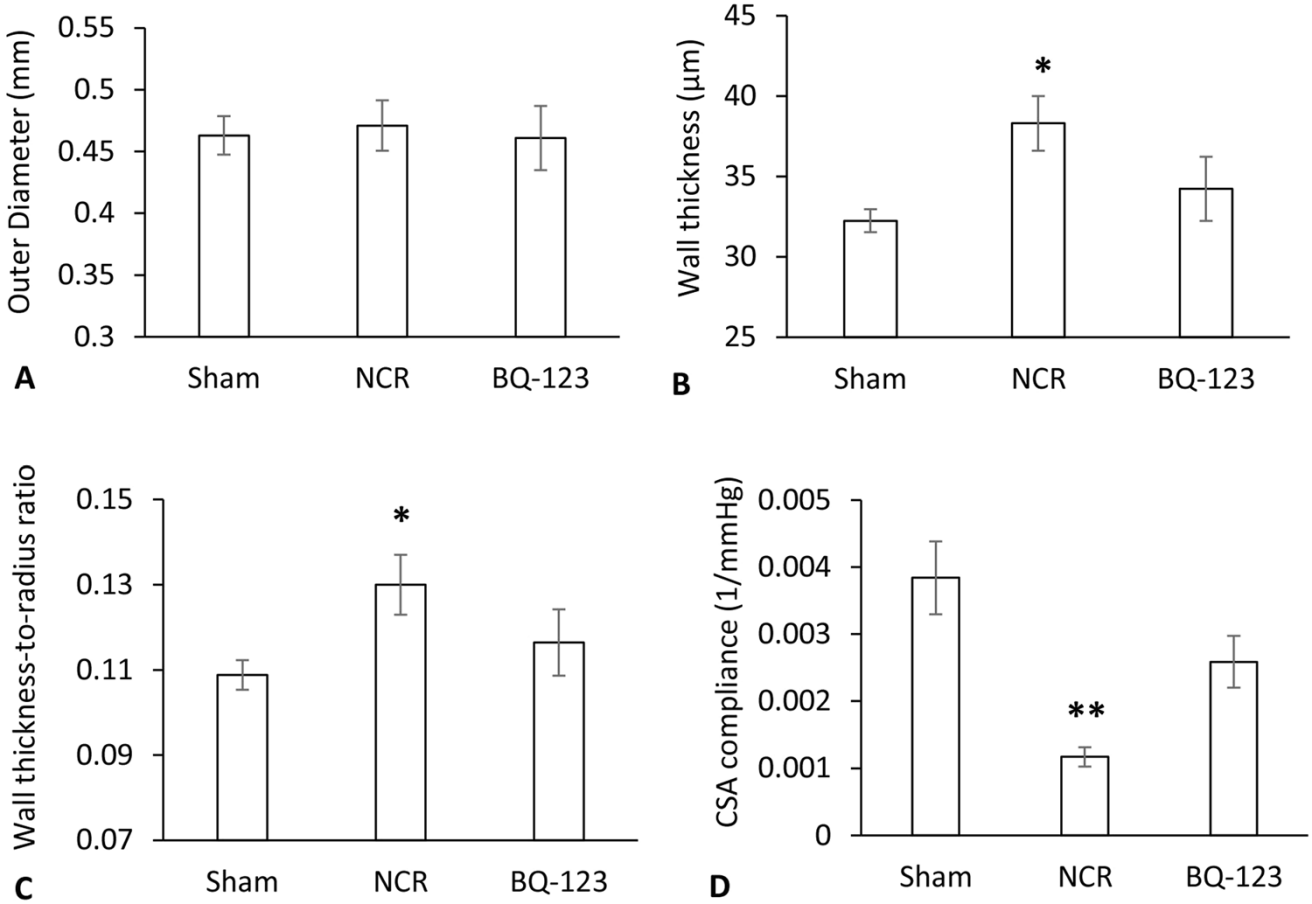
Outer Diameter (**A**), wall thickness (**B**), wall thickness-to-radius ratio (**C**) and normalized cross-sectional area (CSA) compliance (**D**) of peripheral small arteries for sham, aortic NCR and BQ-123 treatment groups. Data correspond to mean ± SEM. *P < 0.05, when aortic NCR (n = 10) compared with sham (n = 8) group. **P < 0.05, when aortic NCR compared with sham and BQ-123 (n = 9) groups.

Figure 3 shows outer diameter, wall thickness (WT, intima-media thickness) and wall thickness-to-radius (WTTR) ratio of peripheral small arteries for sham, NCR and BQ-123 treatment groups. Since the mean diameter is around 460 μm, the arteries used for the study were considered to be distributing muscular arteries (connect conductance and resistance arteries). There were no changes in outer diameter of the peripheral small arteries in sham, NCR and BQ-123 groups. The WT and WTTR in NCR group were greater than that in sham (P < 0.05, paired *t-*test). Although WT and WTRR after BQ-123 treatment have a downward trend as compared to NCR group, the difference is not statistically significant. Compliance of arteries is defined as the change in luminal dimension (diameter, cross-sectional area, or volume) divided by the corresponding change in pressure. We present the compliance as the pressure-cross-sectional area, P-CSA relationship of the peripheral artery. The normalized CSA compliance is used to remove the effect of size so that comparison can be made between different size vessels. Figure 3D shows the normalized CSA compliance in small peripheral arteries after aortic NCR and BQ-123 treatment. The normalized CSA compliance was significantly lower in NCR than that in sham (P < 0.01, paired *t-*test). Although BQ-123 treatment significantly increased the CSA compliance compared with NCR group (p = 0.02, paired *t-*test), it did not fully restore the value when compared to sham (p = 0.06, paired *t-*test)

**Figure 4:**
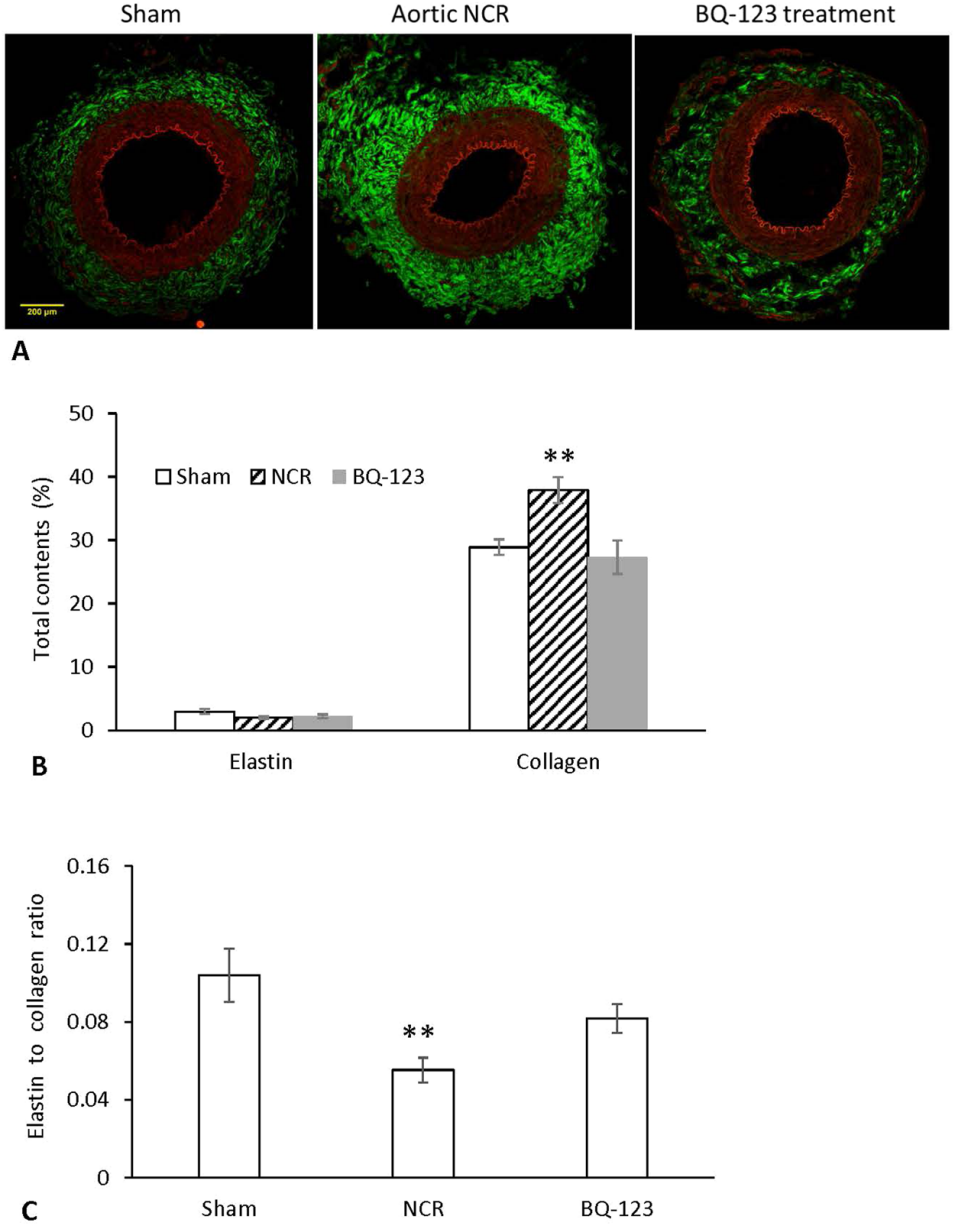
(**A**) MPM images of elastin and collagen fibers of peripheral small arteries for sham, aortic NCR and BQ-123 treatment groups. Red color corresponds to elastin which is restricted to fenestrate internal elastic lamina and external elastic lamina. Green color corresponds to collagen which is mainly located in adventitia; (**B**) Elastin and collagen contents and (**C**) elastin to collagen ratio in peripheral small arteries for sham, aortic NCR and BQ-123 treatment groups. Since the error bar for elastin content is much smaller than that of collagen, it is not visible in the figure. Data correspond to mean ± SEM. **P < 0.05, when aortic NCR (n = 10) compared with sham (n = 8) and BQ-123 (n = 9) groups.

Figure 4A shows Multiphoton microscopy (MPM) images of elastin and collagen fibers of peripheral small arteries for sham, aortic NCR and BQ-123 treatment groups. Red color-coded images correspond to elastin at 520 nm (TPEF signal) and green color-coded images correspond to collagen at 415 nm (SHG signal). The quantitative analysis showed that the total collagen content of peripheral arteries was greater in NCR than that in sham (p < 0.05, paired *t-*test), while the collagen content following BQ-123 treatment was returned to normal levels (Fig. 4B). No difference in total elastin contents was observed between NCR, BQ-123 treatment and sham groups. The elastin to collagen ratio was found to be significantly increased in NCR as compared to sham, but the value was restored to normal levels as well after 3 weeks of treatment with BQ-123 (Fig. 4C).

**Figure 5:**
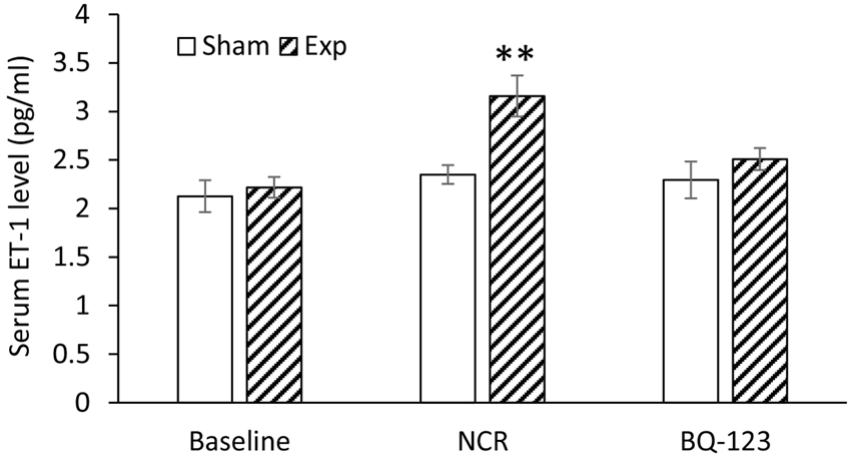
Serum ET-1 levels before and after aortic NCR and BQ-123 treatment for experimental (Exp) and sham groups. Data correspond to mean ± SEM. **P < 0.05, when aortic NCR (n = 10) compared with sham (n = 8) and BQ-123 (n = 9) groups.

Figure 5 shows serum ET-1 levels before and after aortic NCR and BQ-123 treatment. The ET-1 level was significantly increased after aortic NCR compared with sham (P < 0.05, paired *t-*test). With the BQ-123 treatment, the value was restored to pre-intervention level, which was not significantly different from the sham.

**Figure 6:**
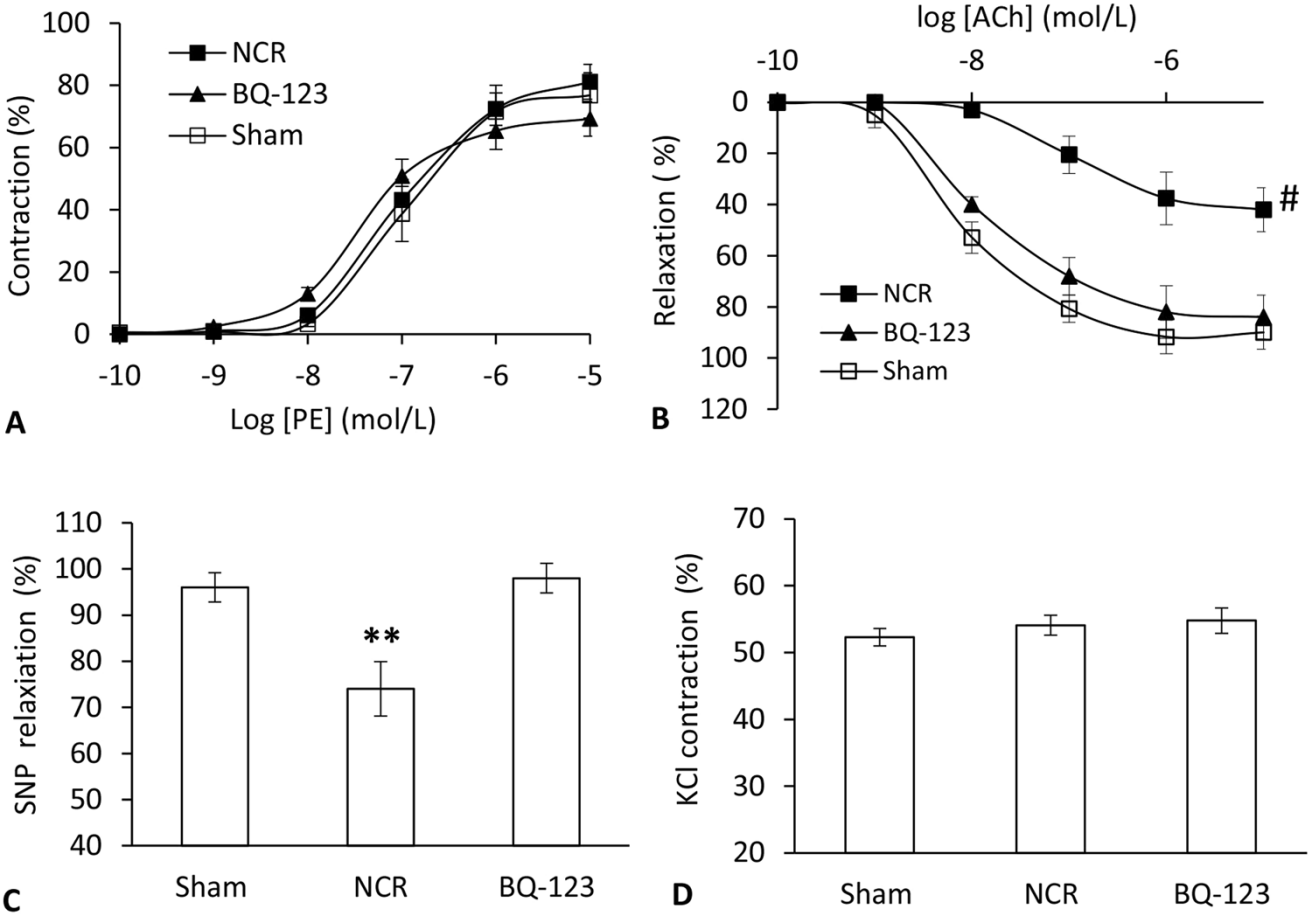
(**A**) Vascular contraction to phenylephrine (PE) and (**B**) endothelium-dependent vasodilation to acetylcholine (ACh) and (**C**) maximal responses of vasodilation to sodium nitroprusside (SNP) and (**D**) contraction to KCl at 60 mmol/L in peripheral small arteries after aortic NCR and BQ-123 treatment. Data correspond to mean ± SEM. ^#^P < 0.05, statistical difference of the dose-dependent curve when aortic NCR (n = 10) compared with sham (n = 8) and BQ-123 (n = 9) groups. **P < 0.05, when aortic NCR compared with sham and BQ-123 groups.

Endothelial function was evaluated by *ex vivo* phenylephrine (PE) pre-contractile endothelium-dependent vasorelaxation. The contractions to PE were similar in sham and aortic NCR rats, and the treatment with BQ-123 did not affect this response (Fig. 6A), exhibiting two similar dose-response curve parameters, E_max_ (the maximum effect) and pED_50_ (the dose causing half-maximal relaxation or contraction) for all experimental groups (Table 2). The endothelium-dependent vasodilation in response to acetylcholine (ACh) is shown in Fig. 6B. Compared to sham, a significant compromised vasodilation to ACh was observed in aortic NCR group (p < 0.05, 2-way ANOVA), but the impaired response was totally recovered after BQ-123 treatment (p < 0.05, 2-way ANOVA). These results were verified by the decreased E_max_ and pED_50_ values following NCR and the increased E_max_ and pED_50_ values following BQ-123 treatment (p < 0.05, paired *t*-test, Table 2). The maximal responses of endothelium-independent vasodilation to sodium nitroprusside (SNP) at 10^−5^ mol/L is shown in Fig. 6C. Sham rats showed an improved relaxation (96%) to SNP than rats with aortic NCR (74%, p < 0.05, paired *t-*test). Similarly, BQ-123 treatment completely restored the relaxation (98%) to SNP. There was no significant difference in vascular contraction to potassium chloride (KCl) at 60 mmol/L among the 3 groups (Fig. 6D).

**Table 2:** E_max_ and pED_50_ for acetylcholine and phenylephrine concentration-response curves in peripheral small arteries.

Agents	Sham	Aortic NCR	BQ-123 treatment
Acetylcholine			
E_max_ (%)	8.0 ± 0.06	6.9 ± 0.07*	7.7 ± 0.10
pED_50_	91.5 ± 4.7	45.6 ± 4.9*	88.5 ± 7.9
Phenylephrine			
E_max_ (%)	7.0 ± 0.16	6.9 ± 0.21	7.3 ± 0.19
pED_50_	76.2 ± 10.5	81.5 ± 10.9	72.4 ± 6.3

Values are mean ± SEM. pED_50_ values are expressed as negative log mol/L. *P < 0.05, when compared to sham and BQ-123 groups (paired *t*-test).

## Discussion

A non-constrictive restraint (NCR) created aortic stiffening (evidenced by elevated PWV and PP) in a rat model which leads to hypertension after 12 weeks. An increase in aortic stiffness caused a significant increase in systemic blood pressure and a significant change in peripheral blood flow pattern. The hypertrophic structural remodeling of peripheral small arteries was observed coincident with decreased arterial compliance and impaired endothelial function. Chronic ET_A_ receptor blockade partially reversed peripheral arterial hypertrophy but completely restored local blood flow and endothelium function, and consequently decreased blood pressure to normotensive values.

There is substantial evidence that PP and PWV provide a measure of arterial stiffness and predict cardiovascular morbidity and mortality^1,28^. In this study, 12 weeks of aortic NCR resulted in a significant increase in PP (23% in central and 24% in peripheral) and MAP (18% in central and 20% in peripheral), reflecting both an increase in SBP and DBP (Table 1). In line with the widened PP, a marked increase in aortic PWV was observed following aortic NCR. Interestingly, we found that short term (4 weeks) of aortic NCR only increased central MAP by 8.8% in our previous study with the same animal model^29^. In comparison, a progressive increase in MAP occurred from 4 week (8.8%) to 12 weeks (18%) of aortic NCR in this study. This result suggests a temporal relationship between aortic stiffening and the development of hypertension. Our finding is also similar to a recent study by Weisbrod *et al*.^8^ who demonstrated that arterial stiffness precedes hypertension in an animal model of diet-induced obesity. Furthermore, the rats with aortic NCR revealed an increase in LV/HW and LV/BW ratio as compared to sham (Fig. 2), indicative of left ventricular hypertrophy, which may be secondary to the elevation of afterload induced by aortic stiffening.

To test the hypothesis whether aortic stiffening has a direct effect on peripheral vessel structure and function, the flow pattern, mechanical and structural property of peripheral small arteries were evaluated. Regional blood flow is thought to be an important regulator of vascular function and structure. Pulsatile flow produces tangential shear stress on arterial endothelium, whereas mean flow contributes to tissue perfusion^14,30^. In the current study, the flow waveform of peripheral small arteries displayed a biphasic pattern, including forward flow toward the lower extremities during systole and reverse flow toward the femoral artery during diastole (Fig. 1A). Aortic NCR caused a pronounced decrease in peripheral mean flow and forward flow, but an increase in peak reverse flow and R/F ratio (Fig. 1B). During diastole, increased reverse flow means more blood flowing back to femoral and less blood going into low extremities. Our data confirmed that increased aortic stiffness can markedly reduce not only systolic but also diastolic flow into the lower extremities, which provides an *in vivo* evidence that aortic stiffening increases pulsatile hemodynamic forces that may be detrimental to the peripheral microcirculation.

WSS is determined by blood flow, vessel geometry and fluid viscosity. Steady WSS is a determinant of normal vascular function through its interaction with endothelial cells^31^. The presence of low shear stresses is frequently accompanied by unstable flow conditions^32^. With the dramatic alteration in peripheral flow patterns following aortic NCR, a pronounced drop in WSS was observed because of the significantly reduced mean flow (Fig. 1C). Since low WSS has been identified as a local risk factor in arterial remodeling and atherogenesis^31,33^, it is possible that adverse effects of aortic stiffening on peripheral WSS may trigger cellular proliferation mechanisms and activate vascular structural and functional remodeling.

Vascular remodeling is believed to be an adaptive process in response to chronic changes in hemodynamic conditions during aging and vascular pathologies^34,35^. With aortic NCR in this study, peripheral small arteries exhibited signs of structural remodeling, characterized by intima-media thickening and increased WTTR ratio. This remodeling was described as inward hypertrophic remodeling due to an increased WTTR ratio but with no substantial changes in outer diameter (Fig. 3). Consistent with the hypertrophic remodeling, arterial compliance (a surrogate marker of arterial elasticity) was found to be significantly reduced, meaning an impaired peripheral arterial elasticity (Fig. 3D). This result suggests a progressive increase in stiffness occurred from central to peripheral arteries during the 12 weeks of aortic NCR, indicating a relationship between the widened PP and the abnormal mechanical property of peripheral arteries through a low WSS effect on vessel wall.

It has been reported that increased wall stiffness of resistance arteries is associated with an increased volume density of collagen, an increased collagen-elastin ratio, or both^36^. Elastin and collagen represent the major distensible and nondistensible component in vessel wall, respectively, whose ratio affects vascular compliance^36,37^. Here, collagen in periphery small arteries was distributed along the vascular wall with higher deposition in the adventitial layer (Fig. 4A). The total amount of collagen was significantly increased whereas elastin did not change after aortic NCR, and hence a greater collagen-elastin ratio. This implies that the influence of increased aortic stiffness on peripheral arterial compliance may, at least in part, attribute to an imbalance between elastin and collagen synthesis.

Elevated plasma levels of ET-1 are correlated with various cardiovascular pathophysiological states, such as incidence of hypertension, heart failure, and severity of left ventricular hypertrophy^18–20,38^. In agreement with our previous study^29^, we confirmed that serum ET-1 level increased after aortic NCR, and the blockade of ET_A_ receptor with BQ-123 treated this increase (Fig. 5). When assessing the effect of blockade of ET_A_ receptor on blood pressure and blood flow, we observed that 3-week BQ-123 treatment partially reversed the increase in PWV and PP, and significantly lowered MAP. Moreover, this decrease in MAP was accompanied by a total recovery of the peripheral blood flow (increased forward flow and decreased reverse flow) and hence the WSS. The present data shows that ET_A_ receptor blockade can reverse hypertension and peripheral blood flow, which appears to be independent of sustained aortic stiffening. The remained high in PP and PWV following the treatment may be related to irreversible deterioration of aortic wall structure, as confirmed by our earlier work^29^. The beneficial effect of the chronic ET_A_ receptor blockade on peripheral blood flow may have important implications for improving local tissue perfusion and end-organ function. In addition, we found that BQ-123 treatment did not attenuate the LV hypertrophy, expressed by unchanged high ratios of LV/HW and LV/BW. Although BQ-123 largely normalized the systemic blood pressure, the persistent aortic stiffening (increased aortic PWV) may lead to a continual elevated afterload, therefore inducing the compensatory myocardial remodeling of LV. This result is consistent with a clinical study by Anand *et al*.^39^, who reported that 6 months of ET_A_ receptor blockade reduced blood pressure, but failed to improve LV hypertrophy in patients with heart failure. Although the underlying mechanisms remain unclear, the renin-angiotensin systems and some growth factors such as platelet-derived growth factor may participate in the development of cardiac remodeling^39^.

It is generally recognized that ET-1 stimulates cell proliferation and acts as a co-mitogen for vascular smooth muscle cells (VSMC) with other growth factors^40,41^. Hypertrophic remodeling of resistance arteries seems to occur in models associated with an upregulated ET system^23,24^. In the present study, with the inhibition of serum ET-1 level by the ET_A_ receptor antagonist, we found that the BQ-123 treatment tended to reduce (although did not reach statistical significance) the increased media thickness and WTTR ratio in peripheral small arteries. Consistent with the attenuated progression of vascular hypertrophy, the arterial compliance was found to be largely recovered following the treatment. Interestingly, however, we found that the BQ-123 treatment markedly reduced collagen content and caused a total recovery of the elastin to collagen ratio in peripheral arteries. Based on this result, we speculate that collagen synthesis is not the only major element which affects vascular compliance and structure. Other mechanisms such us VSMC number, size or both as well as deposition of extracellular protein may be involved in structural and mechanical remodeling. Taken together, our findings demonstrate that the  attenuated progression of peripheral artery hypertrophy and, thereby, the partial recovery of arterial compliance by ET_A_ receptor antagonist may help to restore the peripheral blood flow and WSS, eventually resulting in normalization of systemic blood pressure, despite sustained elevation in aortic stiffness.

The vascular endothelium plays a pivotal role in the regulation of vascular tone and the maintenance of cardiovascular homeostasis by the release of vasoactive factors such as nitric oxide (NO) and ET-1^42^. The normal endothelium can sense WSS and modulates local blood flow^43^. A human study has shown that low flow-mediated shear stress impairs endothelium-dependent vasodilation in peripheral arteries^44^. With the decreased WSS following aortic NCR, we found that the endothelium-dependent relaxations to ACh were markedly blunted (Fig. 6B), meaning impaired endothelium function. For the endothelium-independent relaxations to NO donor, SNP, we found that the maximal response (at dose 10^−5^ mol/L) was significantly decreased with aortic NCR, implying impaired vascular smooth muscle sensitivity to NO (Fig. 6C). Our findings suggest that aortic stiffening-induced hemodynamic changes led to compromised peripheral endothelial function, which may contribute to the onset and progression of hypertension. To further evaluate the role of ET-1 in peripheral endothelial dysfunction through receptor antagonist studies, we found that the BQ-123 treatment can nearly completely normalize impaired endothelium-dependent relaxation to ACh. Moreover, the maximal relaxation of vascular smooth muscle to SNP was improved as well. This is in line with a previous study^45^ showing that blockade of ET_A_ receptor facilitates the maintenance of vasodilation in a hypertension rat model. In addition, we found that the vascular contraction in response to PE remained unaffected in all groups, indicating the sustained expression and transduction of adrenergic receptor following aortic NCR and BQ-123 treatment. Our results support a role of endogenous ET-1 as an important vascular mediator contributing to endothelial functional and structural remodeling. Further studies are needed to investigate multiple signaling pathways of ET-1 receptors in the pathogenesis of aortic stiffening-induced hypertension.

In summary, the current study shows that an increase in aortic stiffness arising from a non-constrictive aortic restraint leads to the development of hypertension through secondary effects on peripheral vasculature. Aortic stiffening-induced hemodynamic, structural and functional changes of peripheral small arteries are associated with increased ET-1 release in the course of hypertension. The full restoration of blood pressure and local blood flow after chronic ET_A_ receptor blockade may be mediated by the improvement of peripheral endothelium function and regression of arterial hypertrophy, albeit its protective effect seems to be independent of aortic stiffness. Our findings establish ET-1 as an early participant in aortic stiffening-induced hypertension and suggest that further exploration of ET_A_ receptor blockade may provide a new strategy for the treatment of hypertension and associated vascular complications.

### Clinical Relevance

The aortic NCR model used to induce aortic stiffening and consequently hypertension has some clinical relevance. Since 1990’s, abdominal and thoracic endovascular aneurysm repair (EVAR and TEVAR) using endograft has gained acceptance as a minimally invasive surgery in selected patients^46,47^. Endograft clearly does not have the normal compliance of aorta and hence inherently increases the stiffness of the aorta. In fact, some clinical studies have demonstrated that endoluminal repair with endografts increases aortic stiffness by measuring carotid-femoral PWV^48–50^. Moreover, repair of coarctation tends to increase aortic stiffness and causes vessel dysfunction, which leads to elevation of blood pressure^51,52^. Therefore, the findings in this study not only advance the basic knowledge of relation between aortic stiffening and hypertension but may also provide valuable clinical feedback to improve the design of endograft (e.g., endograft with aorta-like compliance) that may prevent some of undesirable long-term side effects of aortic stiffening devices.

## Methods

### Animal preparation

Twenty seven Wistar rats at age of 17–18 weeks were randomly divided into three groups. Group 1 (n = 10) underwent aortic NCR for 12 weeks and was terminated at the end of 12 weeks. Group 2 (n = 9) underwent aortic NCR for 12 weeks, then received continuous BQ-123 infusion for 3 weeks. The animals were terminated at the end of 15 weeks. Group 3 (n = 8) was used as the sham-operated control for groups 1 and 2. All animal experiments were performed in accordance with national and local ethical guidelines, including the Institute of Laboratory Animal Research guidelines, Public Health Service policy, the Animal Welfare Act, as approved by Institutional Animal Care and Use Committee at California Medical Innovations Institute, San Diego.

### Surgical procedures

#### Pressure and flow measurement

Animals were anesthetized with 1–2% isoflurane by air inhalation. A pressure catheter (Mikro-tip SPR-407, Millar Instruments, Houston, TX,) was inserted in the aortic arch (proximal site) via the right carotid artery. Heparin (200 U/ml) was used to prevent blood clots in the vessels. Another pressure transducer (Mikro-tip SPR-671) was advanced retrogradely into the abdominal aorta (distal site) via the right femoral artery. The central and peripheral aortic blood pressure waveforms were recorded from these two locations simultaneously during the procedure. A branch (400~500 μm in diameter) of the left femoral artery was exposed carefully. A flow probe (0.5 mm ID) connected to a flow meter (Transonic systems, Ithaca, NY) was then placed around it and local flow rate was recorded for at least 30 minutes. Following the measurement of blood pressure, the small cuts for cannulation on the carotid and femoral arteries were repaired by 11–0 sutures to restore flow.

#### Aortic NCR (glue coating)

A laparotomy (about 3.0 cm) was performed. The distal abdominal aorta between renal and common iliac artery was carefully exposed and tissue glue (cyanoacrylate formulation, Dermabond, Ethicon, NJ) was coated over a length of the aorta. After allowing 5–10 minutes for the glue to harden, a stiff coating formed and covered the anterior and bilateral sides of the aorta with an axial length of 3.0–3.5 cm. The sham group underwent an identical surgical procedure, but without application of glue on the aorta (i.e., the same amount of glue was left near the aorta area with no direct contact with the aorta).

#### BQ-123 treatment

Rats in group 2 received continuous BQ -123 infusion (1 mg/kg/day) for 21 days through Osmotic minipump implantation after 12 weeks of aortic NCR. BQ-123 (Peptides International Inc., Louisville, KY) was dissolved in saline containing 0.5% dimethyl sulphoxide (DMSO). The Osmotic minipump (model 2002, Durect Corporation, CA) was subcutaneously implanted on the side of abdomen in rats. Sham rats received continuous saline infusion through Osmotic minipumps for 21 days.

#### Terminal study

After measurement of pressure and flow from a branch of femoral artery, water-resistant carbon particles were used to mark the same vessel segment (400~500 µm in diameter) to measure axial changes as described in a publication by Guo *et al*.^53^. The external geometry of the arterial segment was photographed to obtain the outer diameter and *in vivo* axial length with the aid of a dissecting microscope. The arterial segment was than harvested for endothelial function and mechanical testing and histological analysis. The heart was harvested to calculate the wet weight.

### Endothelial function

An isovolumic myograph recently developed by our group was used to evaluate the endothelium-dependent vasorelaxation^54^. The small peripheral arterial segment was cannulated on both ends in a physiological bath with HEPES physiologic saline solution (HEPES-PSS, concentration in mmol/l: 142 NaCl, 4.7 KCl, 2.7 sodium HEPES, 3 HEPES acid, 0.15 NaHPO_4_, 1.17 MgSO_4_, 2.79 CaCl_2_, and 5.5 glucose, solution gassed by 95% O_2_ plus 5% CO_2_) and stretched to *in situ* length. The pressure and external diameter were measured with a pressure transducer (Mikro-Tip SPR-524; Millar Instruments) and a digital diameter tracking (DiamTrak v3+ ; Australia), respectively. The vessel segment was pre-constricted with phenylephrine (PE) by a series of doses (10^−10^ to 10^−5^ mol/L in the PSS), and then relaxed with acetylcholine (ACh) by a series of doses: 10^−10^ to 10^−5^ mol/L. The endothelium-independent relaxation to sodium nitroprusside (SNP, 10^−5^ mol/L) was measured to verify the sensitivity of vascular smooth muscle to NO. The overall contractility of vessel was tested with potassium chloride (KCl) at 60 mmol/L. Contraction was expressed as percentage of the response to KCl. Relaxation was expressed as percentage of pre-contraction to PE.

### Mechanical tests

The peripheral arterial segment was cannulated on both ends and fully relaxed in Ca^2+^ free HEPES-PSS. The arterial segment was preconditioned with five cyclic changes in pressure from 0 to 140 mmHg. The pressure was then increased in 20 mmHg step increments from 20 to 140 mmHg in a staircase manner. The passive pressure-diameter relation was recorded. After the mechanical testing, the vessel segment was cut transversely into three or four rings. Each ring was photographed in the no-load state and then cut radially by a scissor to reveal the zero-stress state. The cross section of each sector was photographed 30 minutes after the radial cut (details in Guo *et al*.^53^). The morphological measurements of the *in vitro* axial length, inner and outer circumference, wall thickness (WT), and area in the no-load and zero-stress state were made from the images using a morphometric analysis system (SigmaScan).

### Hemodynamic and mechanical analysis

PWV calculation is based on the difference in arrival times of the pressure wave at the proximal (aorta arch) and distal (abdominal aorta) locations. Since the pressure transducer is visible on radiographs, the propagation distance between the proximal and distal sites was obtained by imaging the animal with the two transducers implantation under an X-ray machine (Philips Fluoroscopy System, picture not shown). PWV (m/s) was calculated by dividing the propagation distance by the difference between the two arrival times (transit time).

The reverse to forward flow ratio (indicative of the extent of peripheral flow reversal) in peripheral small arteries was calculated as: R/F ratio = Q_rev_/Q_fwd_ × 100 (%), where Q_rev_ and Q_fwd_ are the reverse and forward peak flow rate, respectively.

The loaded inner radius of the vessel was determined from the incompressibility assumption. The incompressibility condition for a cylindrical vessel can be expressed as:

1a $${r}_{i}=\sqrt{{r}_{o}^{2}-\frac{{A}_{o}}{\pi {\lambda }_{z}\,}}$$

where r_o_ and r_i_ are the outer and inner radii at the loaded state, respectively. λ_z_ = l/l_o_ is the stretch ratio in the axial direction where l and l_o_ are the vessel length in the loaded and no-load state, respectively and A_o_ is the wall area in the no-load state. The WT at the loaded state was computed as the difference between the outer and inner radius of the vessel as:

1b $$WT={r}_{o}-{r}_{i}={r}_{o}-\sqrt{{r}_{o}^{2}-\frac{{A}_{o}}{{\pi }{{\lambda }}_{z}\,}}$$

where r_o_, A_o_ and λ_z_ were measured quantities.

The wall shear stress (WSS) can be evaluated if assuming a laminar, incompressible Newtonian flow through a rigid cylindrical vessel as given by the following equation:

2 $$WSS=\frac{32uQ}{{\pi }{D}^{3}}$$

where Q and D represent the volumetric flow rate and inner diameter of vessel and µ denotes the viscosity of blood which was assumed to be a constant value of 4 cP.

The volume compliance (C_V_) of the artery was determined by the slope of the pressure-volume relationship; i.e., C_V_ = ∆V/∆P. The lumen cross-sectional area (CSA) was computed from the lumen diameter (D), as CSA = πD^2^/4. The normalized CSA compliance (C_CSA_) was determined as C_CSA_ = ∆CSA/(∆P•CSA) at the physiological pressure as described in our previous publication^29^.

### Elastin and collagen contents

The elastin and collagen in small peripheral arteries were imaged by using a Multiphoton microscope (MPM) as described by Huan *et al*.^55^. Briefly, the arterial segment was fixed with 4% paraformaldehyde in phosphate buffer for 4 hours. The vessel was then transferred to a cryomold containing OCT embedding medium, and frozen in liquid nitrogen. Frozen transverse sections (7 µm) were cut onto glass slide and visualized by the MPM with a combined SHG/TPEF setup (Zeiss LSM 710 NLO). Serial optical sections were simultaneously captured by using the 520 nm line for elastin and the 415 nm line for collagen. All the images were taken under identical conditions of laser intensity, brightness, and contrast. Fluorescence intensity values were used as estimates of elastin and collagen concentration and quantitatively analyzed by ImageJ.

### Serum ET-1 level

Blood samples were collected in ethylenediaminetetraacetic acid (EDTA) tubes for all experimental rats. After centrifugation at 5000 rpm and 4 °C for 15 minutes, serum was immediately separated and stored at −80 °C until analysis. The circulating level of ET-1 in serum was measured by an ELISA kit (R&D system, MN).

### Statistical analysis

Results were shown as mean ± standard error of mean (mean ± SEM). The significance of the differences between two groups was evaluated by either *t*-test or One-way ANOVA. For each dose-response curve to agents, the maximum effect (E_max_) and bolus dose that produced half-maximal relaxation or contraction (expressed as pED_50_) were obtained by fitting the experimental data with a sigmoidal dose response curve. A least-squares fitting function, FindFit (Wolfram Mathematica software, Illinois, USA) was used. Significant differences among the three groups for dose-dependent curves (Fig. 6A and B) were determined by Two-way ANOVA (SigmaStat, California, USA). The results were considered statistically significant when P < 0.05 (2-tailed).

### Data Availability

The datasets generated during the current study are available from the corresponding author on reasonable request.

## References

[1] Vlachopoulos C (2010). Prediction of cardiovascular events and all-cause mortality with central haemodynamics: a systematic review and meta-analysis. Eur Heart J..

[2] Boutouyrie P (2002). Aortic stiffness is an independent predictor of primary coronary events in hypertensive patients: a longitudinal study. Hypertension.

[3] Izzo JL (2004). Arterial stiffness and the systolic hypertension syndrome. Curr Opin Cardiol.

[4] Laurent S (2003). Aortic stiffness is an independent predictor of fatal stroke in essential hypertension. Stroke..

[5] Mattace-Raso FU (2006). Arterial stiffness and risk of coronary heart disease and stroke: the Rotterdam study. Circulation..

[6] Franklin SS, Khan SA, Wong ND, Larson MG, Levy D (1999). Is pulse pressure useful in predicting risk of coronary heart disease?. Circulation.

[7] Mitchell GF (2006). The role of arterial stiffness in the pathogenesis of hypertension and cardiovascular disease. Cardiology Rounds.

[8] Weisbrod RM (2013). Arterial stiffening precedes systolic hypertension in diet-induced obesity. Hypertension.

[9] Kaess BM (2012). Aortic stiffness, blood pressure progression, and incident hypertension. JAMA..

[10] Christensen KL (1991). Reducing pulse pressure in hypertension may normalize small artery structure. Hypertension..

[11] Lund-Johanson P (1983). Haemodynamics in early essential hypertension: still an area of controversy. J Hypertens..

[12] James MA, Watt PA, Potter JF, Thurston H, Swales JD (1995). Pulse pressure and resistance artery structure in the elderly. Hypertension..

[13] Mitchell GF (2005). Cross-sectional relations of peripheral microvascular function, cardiovascular disease risk factors, and aortic stiffness: the Framingham Heart Study. Circulation..

[14] Martinez-Lemus LA, Hill MA, Meininger GA (2009). The plastic nature of the vascular wall: a continuum of remodeling events contributing to control of arteriolar diameter and structure. Physiology.

[15] Najjar SS (2008). Pulse wave velocity is an independent predictor of the longitudinal increase in systolic blood pressure and of incident hypertension in the Baltimore Longitudinal Study of Aging. J Am Coll Cardiol..

[16] Yambe M (2007). Arterial stiffness and progression to hypertension in Japanese male subjects with high normal blood pressure. J Hypertens..

[17] Galie N, Manes A, Branzi A (2004). The endothelin system in pulmonary arterial hypertension. Cardiovasc Res.

[18] Haak T, Jungmann E, Raab C, Usadel KH (1994). Elevated endothelin-1 levels after cigarette smoking. Metab. Clin. Exp..

[19] Kosicka T, Kara-Perz H, Perz S (2006). Evaluation of plasma endothelin-1 concentration in tobacco smoking patients with essential hypertension. Przeglad lekarski..

[20] Rahman MM (2007). Increased vascular contractility in isolated vessels from cigarette smoking rats is mediated by basal endothelin release. Vascul Pharmacol.

[21] Hirai Y (2004). Plasma endothelin-1 level is related to renal function and smoking status but not to blood pressure: an epidemiological study. J Hypertens..

[22] Pollock DM, Keith TL, Highsmith RF (1995). Endothelin receptors and calcium signaling. FASEB J..

[23] Amiri F (2004). Endothelium-restricted overexpression of human endothelin-1 causes vascular remodeling and endothelial dysfunction. Circulation.

[24] Fukuda G (2005). Endothelinmediated remodeling in aortas of diabetic rats. Diabetes Metab Res Rev..

[25] Allahdadi KJ, Walker BR, Kanagy NL (2005). Augmented endothelin vasoconstriction in intermittent hypoxia-induced hypertension. Hypertension.

[26] Opitz CF (2008). Inhibition of endothelin receptors in the treatment of pulmonary arterial hypertension: does selectivity matter?. Eur Heart J..

[27] Pollock DM, Pollock JS (2001). Evidence for endothelin involvement in the response to high salt. Am J Physiol Renal Physiol..

[28] Benetos A, Rudnichi A, Safar M, Guize L (1998). Pulse pressure and cardiovascular mortality in normotensive and hypertensive subjects. Hypertension.

[29] Guo X, Lu X, Yang J, Kassab GS (2014). Increased aortic stiffness elevates pulse and mean pressure and compromises endothelial function in Wistar rats. Am J Physiol Heart Circ Physiol..

[30] O’Rourke MF, Hashimoto J (2007). Mechanical factors in arterial aging: a clinical perspective. J Am Coll Cardiol..

[31] Lu D, Kassab GS (2011). Role of shear stress and stretch in vascular mechanobiology. J. R. Soc. Interface.

[32] Ku DN, Giddens DP, Zarins CK, Glagov S (1985). Pulsatile flow and atherosclerosis in the human carotid bifurcation: Positive correlation between plaque location and low oscillating shear stress. Arteriosclerosis.

[33] Asakura T, Karino T (1990). Flow patterns and spatial distribution of atherosclerotic lesions in human coronary arteries. Circ Res.

[34] Touyz RM (2007). Vascular remodeling, retinal arteries, hypertension. Hypertension.

[35] Heerkens EH (2006). AlphaV integrins are necessary for eutrophic inward remodeling of small arteries in hypertension. Hypertension.

[36] Intengan HD, Thibault G, Li JS, Schiffrin EL (1999). Resistance artery mechanics, structure, and extracellular components in spontaneously hypertensive rats: effects of angiotensin receptor antagonism and converting enzyme inhibition. Circulation..

[37] Sharifi AM, Li JS, Endemann D, Schiffrin EL (1998). Effects of enalapril and amlodipine on small-artery structure and composition, and on endothelial dysfunction in spontaneously hypertensive rats. J Hypertens..

[38] Hua L (2000). Relationship between hypertensive LVH and levels of endothelin and nitric oxide. Hypertens Res..

[39] Anand I (2004). Long-term effects of darusentan on left-ventricular remodelling and clinical outcomes in the EndothelinA Receptor Antagonist Trial in Heart Failure (EARTH): randomised, double-blind, placebo-controlled trial. Lancet..

[40] Moreau P (1997). Angiotensin II increases tissue endothelin and induces vascular hypertrophy: reversal by ET -receptor antagonist. Circulation..

[41] Peifley KA, Winkles JA (1998). Angiotensin II and endothelin-1 increase fibroblast growth factor-2 mRNA expression in vascular smooth muscle cells. Biochem Biophys Res Commun.

[42] Puddu P, Puddu GM, Zaca F, Muscari A (2000). Endothelial dysfunction in hypertension. Acta Cardiol..

[43] Koller A, Huang A, Sun D, Kaley G (1995). Exercise training augments flow-dependent dilation in rat skeletal muscle arterioles: role of endothelial nitric oxide and prostaglandins. Circ Res..

[44] Celermajer DS (1992). Non-invasive detection of endothelial dysfunction in children and adults at risk of atherosclerosis. Lancet.

[45] Barton M (1998). Endothelin ET_A_ receptor blockade prevents increased tissue endothelin-1, vascular hypertrophy, and endothelial dysfunction in salt-sensitive hypertension. Hypertension..

[46] Beebe HG, Cronenwett JL, Katzen BT, Brewster DC, Green RM (2001). Results of an aortic endograft trial: impact of device failure beyond 12 months. J Vasc Surg.

[47] Makaroun MS, Chaikoff E, Naslund T, Matsumura JS (2002). Efficacy of a bifurcated endograft versus open repair of abdominal aortic aneurysms: a reappraisal. J Vasc Surg.

[48] Sekhri AR, Lees WR, Adiseshiah M (2004). Measurement of Aortic Compliance in Abdominal Aortic Aneurysms Before and After Open and Endoluminal Repair: Preliminary Results. J Endovasc Ther.

[49] van Herwaarden JA (2006). Aortic Compliance Following EVAR and the Influence of Different Endografts: Determi nation Using Dynamic MRA. J Endovasc Ther.

[50] Lantelme P (2009). Effect of abdominal aortic grafts on aortic stiffness and central hemodynamics. J Hypertens..

[51] de Divitiis M, Rubba P, Calabrò R (2005). Arterial hypertension and cardiovascular prognosis after successful repair of aortic coarctation: A clinical model for the study of vascular function. Nutr Metab Cardiovasc Dis.

[52] Vriend JWJ, Mulder BJM (2005). Late complications in patients after repair of aortic coarctation: implications for management. Int. J. Cardiol.

[53] Guo X, Lu X, Ren H, Levin ER, Kassab GS (2006). Estrogen modulates the mechanical homeostasis of mouse arterial vessels through nitric oxide. Am J Physiol Heart Circ Physiol..

[54] Lu X (2010). Rosiglitazone reverses endothelial dysfunction but not remodeling of femoral artery in Zucker diabetic fatty rats. Cardiovasc Diabetol.

[55] Chen H (2013). Biaxial deformation of collagen and elastin fibers in coronary adventitia. J Appl Physiol..

